# Rapid and Sensitive SERS Detection of Bisphenol A Using Self-assembled Graphitic Substrates

**DOI:** 10.1038/s41598-017-17030-9

**Published:** 2017-12-01

**Authors:** Pei-Ying Lin, Chiung-Wen Hsieh, Shuchen Hsieh

**Affiliations:** Department of Chemistry and Nanoscience and Nanotechnology, National Sun Yat-sen University, Kaohsiung, 80424 Taiwan

## Abstract

We have prepared and tested a new surface enhanced Raman scattering (SERS) substrate based on self-assembled graphitic sheets to detect bisphenol A (BPA) in plastic consumer goods. Transmission electron microscopy (TEM) and atomic-force microscopy (AFM) were used to characterize the structure of the graphitic sheets and showed a lattice spacing of 0.24 nm and layer height of 0.34 nm. These values were comparable to single monolayer graphene. The effective SERS detection limit of this method is 1 μM BPA, which is lower than the European Union specific migration limit for BPA of 0.6 mg/kg (2.6 μM). When used in salt solutions, graphitic sheets exhibited ultra-sensitivity toward BPA of 0.025 M to 2 M, which was broader than physiological ionic strength (0.14 M) and urinary NaCl (0.17 M). Our results demonstrated that this graphitic sheet based SERS detection platform can be used to determine BPA levels leached from commercial polycarbonate plastic products and for on-site rapid analysis with good results.

## Introduction

Bisphenol a (BPA) is an organic synthetic compound that is used in industry to make plastics and epoxy resins for consumer goods and industrial applications. There is established concern that exposure can lead to a variety of health problems in humans^1–3^. Studies have shown that BPA can leach from containers or container linings then migrate into the food or beverages and be ingested^4,5^ Exposure to BPA (which is known to mimic estrogen), may cause reproduction dysfunction and lead to birth defects in children, breast cancer, recurrent miscarriages, and other ailments^6,7^. The human health hazards related to BPA continue to be investigated, and thus ultra-sensitive methods for detection of low levels of BPA in common household products and foods are of great interest. At present there are several analytical methods used to detect BPA levels in the environment, including high-performance liquid chromatography (HPLC), gas chromatography coupled with mass spectrometry (GC-MS), enzyme-linked immune sorbent assay (ELISA), molecule imprinting techniques, and electrochemical sensors^3,8,9^. In addition, a direct (label-free) immunosensor is becoming popular because it reduces sample preparation time and simplifies the sensing protocol^10,11^.

Surface-enhanced Raman scattering (SERS) is widely used for increasing the Raman scattering signal of molecules adsorbed on rough noble metal, or metal particle decorated substrates^12^. Recently, new types of SERS-active hybrid materials have been reported, such as graphene layers (graphitic sheets), with deposited metal nanoparticles, which can further enhance Raman signal detection^13–16^. Graphene is attractive because it can be produced with a large surface area, good thermal conductivity, high electrical conductivity, and high electron transfer rate^17–19^. Because of these properties, graphene-based Raman scattering is regarded as a versatile characterization tool with ultra-sensitivity for SERS detection^20,21^. BPA, exhibits only a very weak affinity for adsorption on metal surfaces, thus making BPA detection by traditional SERS methods ineffective. Moreover, the complex constituent mixture in “real” samples can interfere with the Raman scattering signal, resulting in poor detection specificity. Thus, research continues with efforts to identify appropriate SERS substrates that have a strong affinity for BPA.

Here we report on the preparation and evaluation of SERS-active (metal-free) graphitic sheet substrates for ultra-sensitive detection of BPA by SERS methods. The graphitic sheets were prepared using silane-based self-assembled monolayers (SAMs) and then characterized using transmission electron microscopy (TEM, HRTEM), energy dispersive X-ray spectroscopy (EDS), and atomic force microscopy (AFM). Surface-enhanced Raman spectroscopy was the core analytical technique used to detect BPA on the graphitic sheet substrates. We believe that this method will allow ultra-sensitive determination of BPA in a wide variety of samples for clinical, industrial, and consumer product research applications.

## Results

### Surface characterization of graphitic sheets

**Figure 1:**
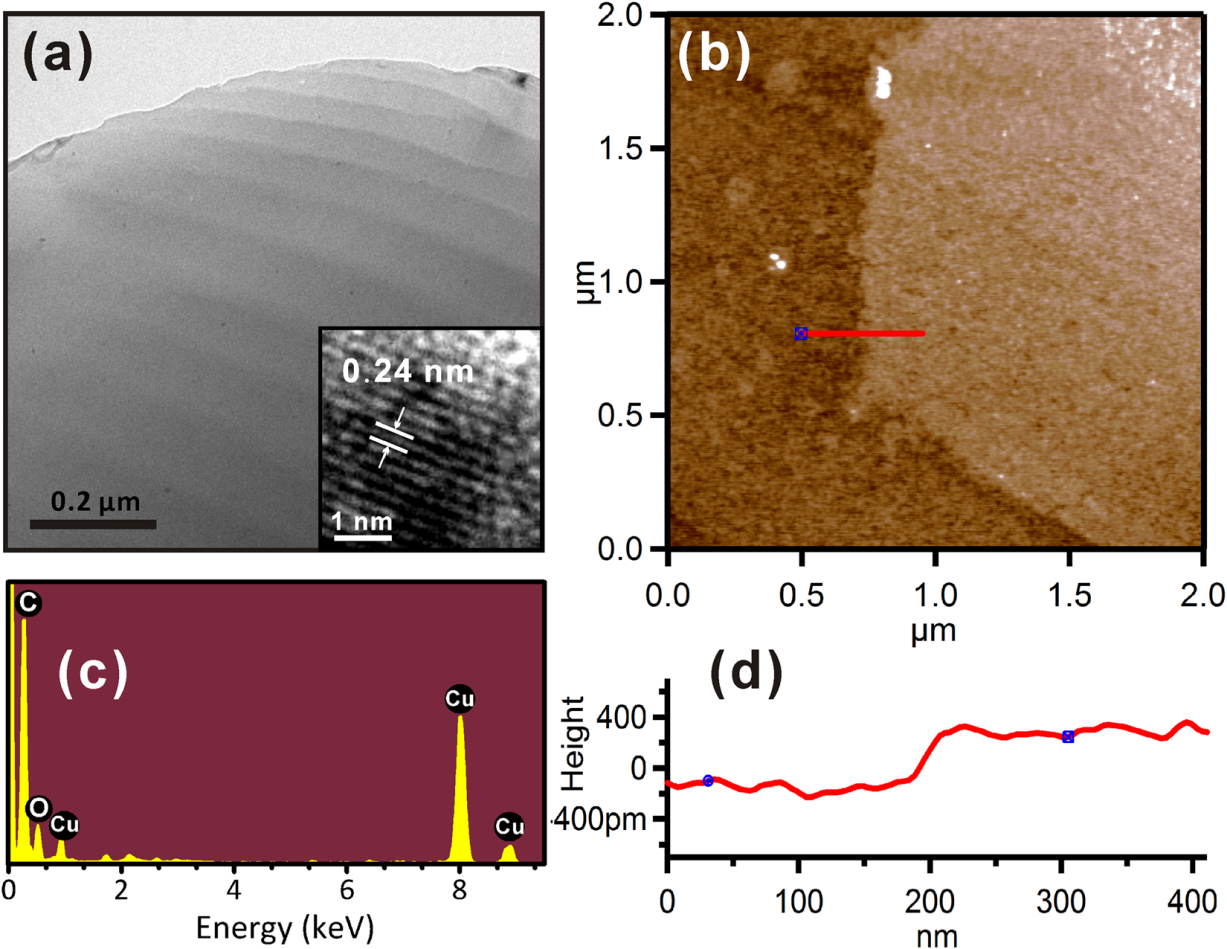
(**a**) TEM and HRTEM (inset) images of graphitic sheet. (**b**) EDS spectrum showing the chemical composition of graphitic sheets (inset shows EDS spectrum of the grid only). (**c**) Surface topography AFM images (2 × 2 μm^2^) and (**d**) line section analysis obtained from graphitic sheet on a silicon substrate.

TEM and HRTEM images in Fig. 1a show that the graphitic sheets had a lattice spacing of 0.24 nm, which is comparable to the lattice constant of graphene^22^. To examine the chemical composition of the graphitic sheets, the EDS spectrum was acquired as shown in Fig. 1b. The primary component of the graphitic sheets was carbon, with a minority contribution from oxygen (~12%) which may be due to oxygen atoms at structural defects^23^. The Cu peaks in the spectra originate from the TEM grid (Fig. 1b inset).

The topographic AFM image and corresponding line scan in Fig. 1c and d show a typical graphitic sheet on a silicon substrate (>1 μm lateral extent). Line scan analysis reveals that the height of the graphitic sheet on silicon is ~0.34 nm, which is comparable to the interlayer spacing of graphene^24^. Thus, the graphitic sheets fabricated in our study exhibit structural characteristics that are nearly identical to those of graphene.

### Determination of feasibility and detection limit

**Figure 2:**
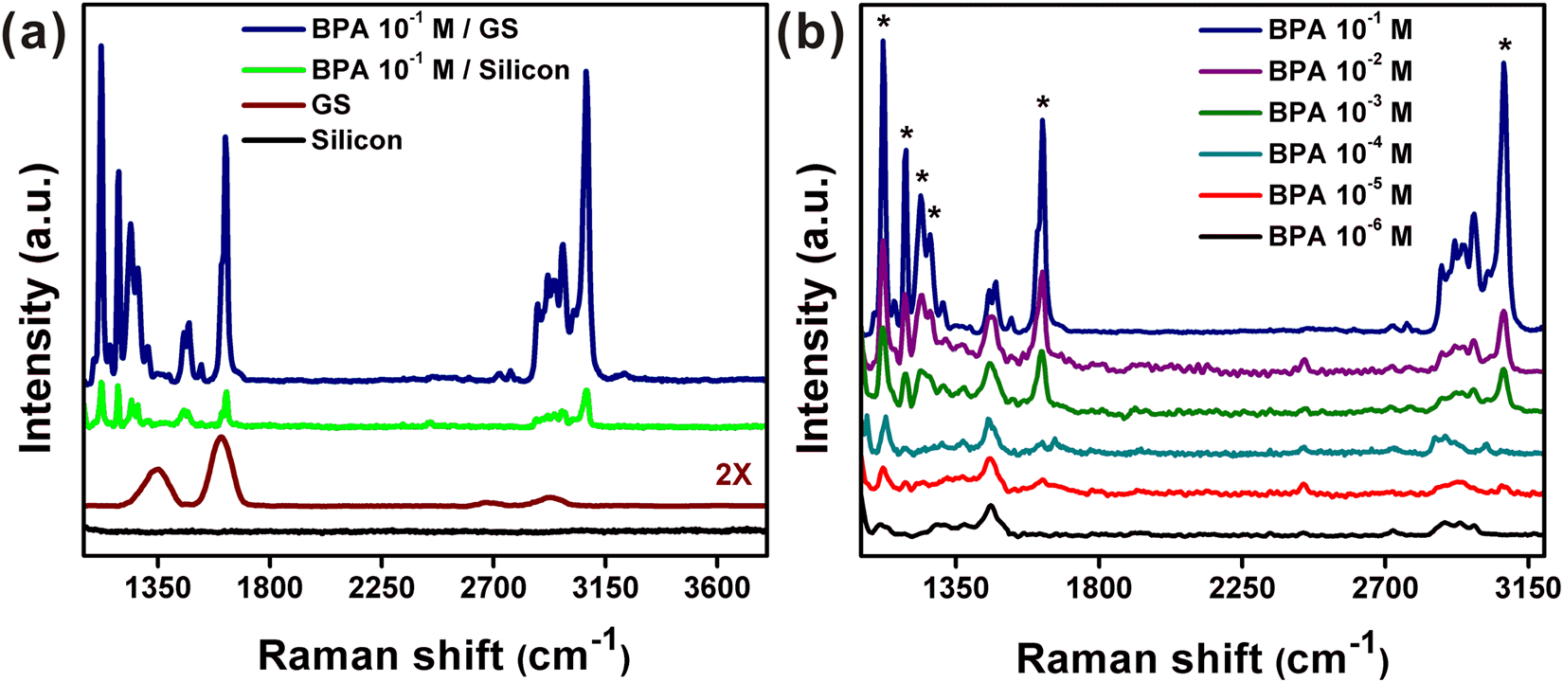
(**a**) Raman spectra of 10^−1^ M BPA deposited on a graphitic sheet substrate and on a bare silicon substrate, respectively. The bottom curves are normal Raman spectra of the same substrates without BPA. The inset shows light scattering images of pure water (*left*) and graphitic sheet solution (*right*) using a 532 nm laser. (**b**) Concentration-dependent SERS spectra of BPA on graphitic sheets adsorbed on a silicon substrate showing a dilution series from 10^−1^ to 10^−6^ M.

The range of products which may contain BPA is broad, thus the capability to detect and monitor BPA in various mixtures is important. We first investigated Raman spectra of a clean silicon substrate and graphitic sheets on silicon, and the SERS spectra of 10^−1^ M BPA deposited onto both substrates separately (Fig. 2a). The inset in the figure shows vials of pure water (*left*) and of the stock graphitic sheet solution (*right*) illuminated using a 532 nm laser. Both vials are optically clear but laser light is scattered in the stock solution. Optical scattering and the Raman result shown in (Fig. 2a – GS), confirm that the stock solution contained graphitic sheets.

No peaks were observed in Raman spectra from a clean silicon substrate. However, Raman spectra from graphitic sheets on silicon had two prominent peaks at 1341 cm^−1^ and 1602 cm^−1^ which are assigned to the D and G bands, respectively. The D band is associated with a breathing mode of the sp^2^ carbon rings and is active when the ring is adjacent to defects, and the G band corresponds to optical E_2g_ phonons^25^. The SERS spectra of 10^−1^ M BPA on both substrates in Fig. 2a exhibited characteristic peaks at 1121 cm^−1^, 1188 cm^−1^, 1240 cm^−1^, 1269 cm^−1^, 1621 cm^−1^, and 3072 cm^−1^. The peaks at 1121 and 1188 cm^−1^ are attributed to the CH wagging vibration (in-plane), and the two weaker peaks at 1240 and 1269 cm^−1^ are attributed to the C–O stretching vibration^26^. The peaks at 1621 and 3072 cm^−1^ are assigned to the phenyl ring stretching and phenyl-hydrogen stretching modes^27^. The peak at 2450 cm^−1^ is attributed to the photoluminescence signal of benzene rings in BPA. While SERS signal was detected for BPA on both substrates, the graphitic sheets on silicon exhibited a very strong SERS enhancement compared to the silicon–only substrate. This may be attributed to π–π stacking between delocalized sp^2^ carbon structures of graphitic sheets and the benzene rings of BPA^15,28,29^.

To confirm the homogeneity of the graphitic sheet substrates for SERS, we performed a BPA (10^−1^ M) Raman mapping experiment. Results from each tested region (I, II, III, IV and V) showed nearly identical BPA SERS signal and demonstrates that the graphitic sheet substrate exhibits good uniformity, as shown in Supplementary Fig. S1. Quantitative detection of BPA on graphitic sheet substrates was performed by measuring the SERS signal intensity of a dilution series of BPA solutions (10^−1^–10^−6^ M) as shown in Fig. 2b. The SERS signal was very strong at 10^−1^ M BPA, and was still detectable even at a concentrations of 10^−6^ M (1 μM), which is lower than the European Union migration limit of BPA in food of 0.6 mg/kg (2.6 μM)^30^. To further analyze these data, we selected the peak at 1121 cm^−1^ as the reference, and plotted the Raman intensity (integrated peak area) versus BPA concentration on a log-log scale for quantitative analysis. Integrated peak calculations were perfomred using OriginPro software. The literature shows that a linear relationship between Log *I* and Log *C* is reasonable as the Raman intensity is proportional to surface coverage of adsorbed molecules^31,32^. Higher molecular concentrations lead to stronger Raman spectra with a linear relationship on a log-log plot. This allows for the calibration of our method to determine unknown concentrations of BPA solutions. Supplementary Fig. S2 shows good linearity between the Log *C* of BPA and Log *I* of the reference peaks (1121 cm^−1^). The linear regression gives a relationship as Log *I* = 5.608 + 0.203 Log *C* with a correlation coefficient (R^2^) of 0.991. This further shows that the detection limit for BPA molecules can reach as low as 1 × 10^−6^ M.

**Figure 3:**
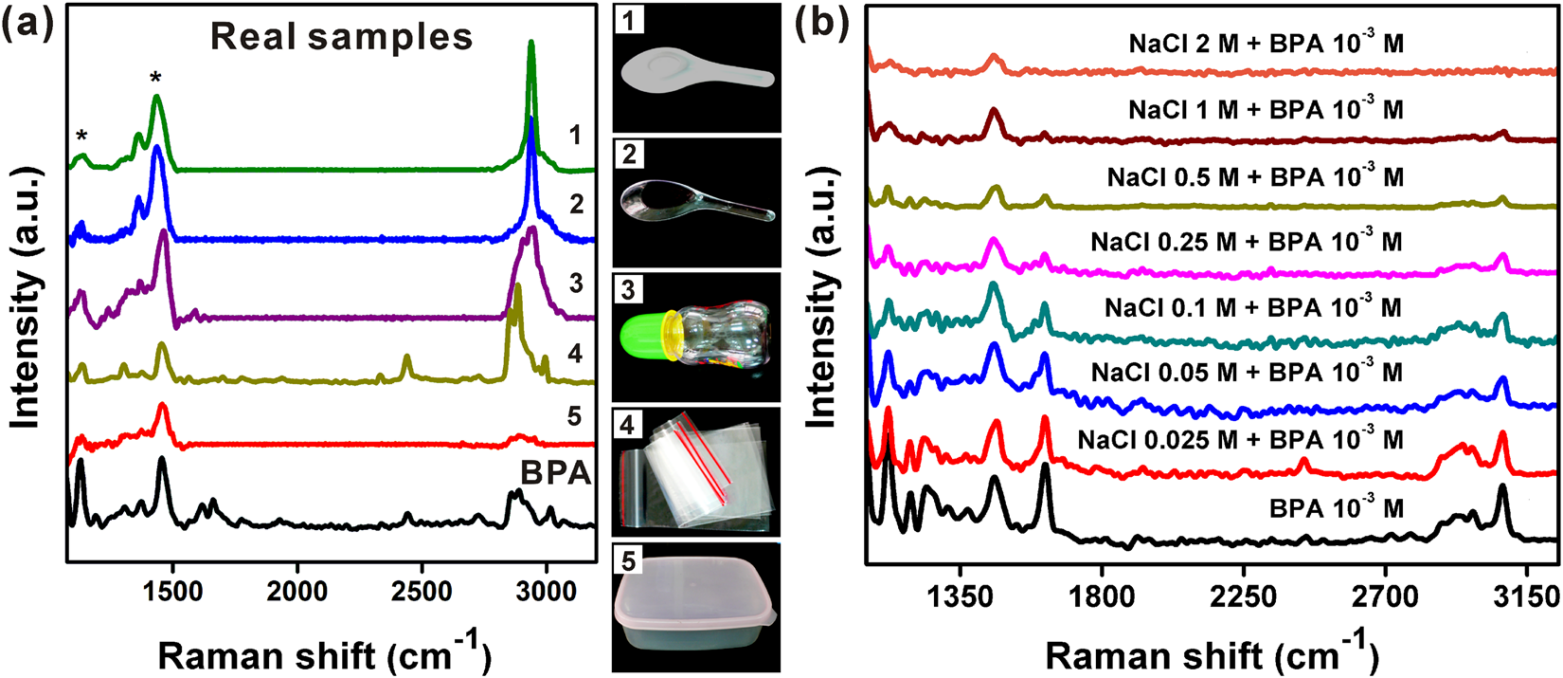
(**a**) Detection of BPA using graphitic sheet substrates presents in daily plastic products by SERS spectra. (**b**) SERS spectra of 10^−3^ M BPA with various concentrations of NaCl using graphitic sheet substrate.

We next examined the utility of this method for detecting BPA leached from “real-world” samples under controlled environmental conditions. Maragou *et al*. suggested that BPA migration can occur when boiled water is poured into polycarbonate baby bottles, with measured BPA concentrations in the liquid ranging from 2.4 to 14.3 mg/kg^4^. Additionally, Nerin *et al*. found BPA in microwavable polycarbonate plastic containers present at a concentration of 30 μg/g in the plastic and estimated migration levels of 6.5 μg/g in foods^33^. In our experiments, plastic spoons (two types), baby bottles, re-sealable zipper storage bags, and airtight food containers (Fig. 3a *right* 1–5) were leach-tested by placing them in contact with 5 mL of boiled water (80 °C) for 1 hour. An aliquot of the collected water was then deposited onto the graphitic sheet substrates for SERS analysis to determine whether BPA had leached from the samples. As shown in Fig. 3a, the characteristic CH wagging peak (in-plane) at ~1121 cm^−1^ was observed for each sample (1–5), at measured concentrations of 1 × 10^−5^, 3.2 × 10^−6^, 2.0 × 10^−5^, 1 × 10^−6^, and 6.3 × 10^−7^ M, respectively. The BPA spectrum at the bottom of the graph is reproduced for reference from the dilution series (Fig. 2) and is 1 × 10^−4^ M BPA. The detection limit of our method was much lower than the typical values measured for consumer products reported by Maragou *et al*. and Nerin *et al*., thus demonstrating that this SERS-based graphitic sheet method for monitoring BPA leached from plastic materials is practical and rapid, as well as quantitative.

### Salt effect

In canned foods, researchers have found that factors such as salt ion concentration can influence the migration of BPA from the internal lining of the can to the contents^34^. Additionally, on graphitic sheet substrates, NaCl may compete for the adsorption sites with BPA^28^. To examine how these factors might influence our method, we measured the SERS signal for 10^−3^ M BPA samples prepared with increasing NaCl concentrations of 0.025 M to 2 M NaCl (Fig. 3b). A decrease in SERS signal intensity with increasing NaCl concentration was observed, which may be explained by the occupation of active sites on the graphitic sheets by NaCl, or by a surface-charge screening effect caused by the higher ionic strength, which could reduce the detection efficiency. However, in clinical applications, BPA is monitored in human serum or urine samples where the physiologic ionic strength of NaCl is 0.14 M and 0.17 M, respectively. Samples at these NaCl concentrations were tested in our experiments (0.025 M to 2 M of NaCl), and BPA was detected, demonstrating the feasibility of the method for BPA detection in body fluids and salt solutions.

## Conclusions

In this work, we report on the development of a SERS-based detection platform for BPA that uses graphitic sheets prepared by SAM methods. A significant Raman signal enhancement was observed for BPA on graphitic sheets, with a detection limit of <1 μM. We applied the method to “real-world” consumer product sample testing (Plastic spoons, baby bottles, re-sealable zipper storage bags, and airtight food containers) and were able to detect low levels of BPA in every sample, indicating that leaching had occurred under the conditions of the experiment. In NaCl, we observed good BPA detection sensitivity at NaCl concentrations of 0.025 M up to 2 M which includes NaCl concentrations typically found in body fluids. Our results demonstrate highly sensitive BPA detection, using a simple, label-free, SERS-based platform. This method has potential for applications in clinical, industrial, and consumer product testing research.

## Methods

### Synthesis of graphitic sheets

Graphitic sheets were fabricated from self-assembled octadecyltrichlorosilane (OTS) films on silicon^35^. The OTS films were annealed in a tube furnace (Lindberg/Blue M, model STF55433C–1) for 30 minutes at 300 °C, immersed into a micro tube with 1-ml pure water, then immediately placed in an ultrasonic bath (Delta DC200H) for 2 minutes (40 kHz ultrasonic frequency, 200 W power output) at room temperature. The resulting supernatant fluid was used directly as the stock solution for all experiments. Throughout the study, Milli-Q reagent-grade (type I) water (18.2 MΩ-cm@25 °C) was used.

### Characterization methods

Characterization of as-prepared graphitic sheets was performed by TEM, EDS, and AFM. Samples for TEM were prepared by first depositing a droplet of stock solution onto a carbon-free, 1000-mesh copper grid (Ted Pella Inc., CA USA) and allowing the sample to dry in air. TEM images were acquired using an EOL JEM-2100, operating at 200 kV. Chemical analysis of the graphitic sheets (using the TEM samples) was carried out by EDS using a JEOL JEM-3010 linked with an eXL-II energy dispersive X-ray analysis system at a resolution of 136 eV FWHM at 5.9 keV. AFM samples were prepared by depositing a droplet of stock solution onto a clean silicon surface at room temperature and allowing them to dry. Topographic AFM images were acquired using an MFP-3D^TM^ AFM (Asylum Research, Santa Barbara, CA, USA) operated in AC “tapping” mode under ambient conditions. A silicon cantilever (Olympus, AC240TS) with a nominal spring constant of 2 Nm^−1^ was used for all images using a scan rate of 1.0 Hz and an image resolution of 512 × 512 pixels.

### SERS measurements

For SERS experiments, samples were prepared by dissolving BPA in ethanol (Echo Chemical, ROC) and then depositing a 2 μl aliquot of a series of concentrations (10^−1^, 10^−2^, 10^−3^, 10^−4^, 10^−5^, 10^−6^ M) on graphitic sheets on silicon, followed by drying at room temperature under ambient conditions. All Raman and SERS experiments were performed on a Raman microscope (WiTec alpha 300 R) with a 532 nm incident laser at 21.9 mW powers. A holographic grating (1800 grooves mm^−1^) and a 1024 × 127 pixel back-illuminated CCD detector with total accumulation times of 30 seconds were used.

## Electronic supplementary material

Supplementary Information
