# Supplementary material for: Rapid and Sensitive SERS Detection of Bisphenol A Using Self-assembled Graphitic Substrates
Source: Sci Rep. 2017 Dec 1;7:16698. doi: 10.1038/s41598-017-17030-9 (PMC5711794; doi:10.1038/s41598-017-17030-9)
Supplement: Supplementary file 1 — Supplementary Information [file 41598_2017_17030_MOESM1_ESM.pdf]

Supplementary Information

Rapid and Sensitive SERS Detection of Bisphenol A Using Self-assembled Graphitic Substrates

Pei-Ying Lin[1], Chiung-Wen Hsieh[1], Shuchen Hsieh[1]

[1]Department of Chemistry and Nanoscience and Nanotechnology, National Sun Yat-sen University, Kaohsiung, 80424, Taiwan.

Correspondence and requests for materials should be addressed to S.H. (email: shsieh@faculty.nsysu.edu.tw)

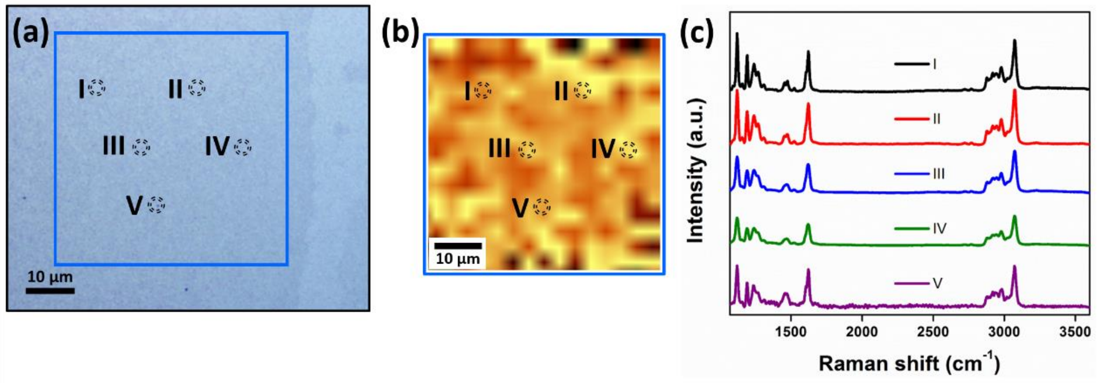

Figure S1. (a) Optical microscope image of graphitic sheet substrate with BPA. (b) Raman mapping image of BPA at 1121 cm^{-1} acquired within the region defined by the blue frame in the optical microscope image (a). (c) Raman spectra of BPA (I, II, III, IV and V) corresponding to each point of the graphitic sheet substrate shown in the Raman mapping image (b).

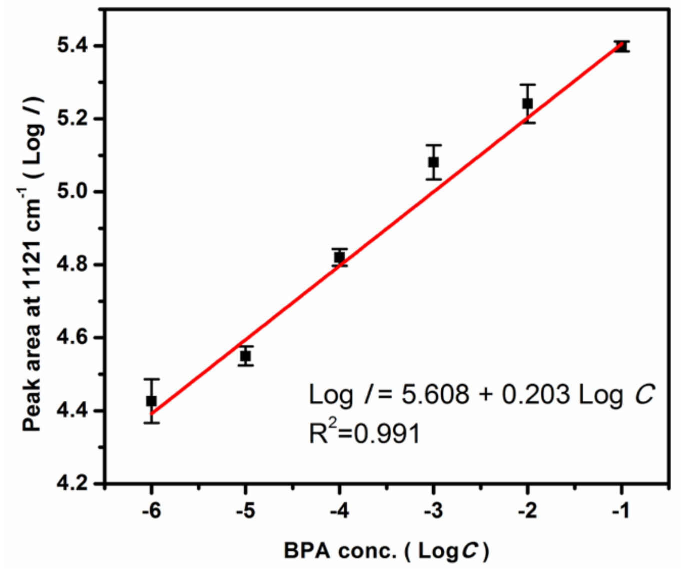

Figure S2. The linear relationship between the log of the integrated intensity (at 1121 cm^{-1}) and the log of BPA concentration. Each spectrum was measured three times.
